# Cooperative Treatment of Gastric Cancer Using B7-H7 siRNA and Docetaxel; How Could They Modify Their Effectiveness?

**DOI:** 10.34172/apb.2023.055

**Published:** 2022-07-02

**Authors:** Nadia Bolandi, Mohammad Hassan Khadem Ansari, Yousef Rasmi, Behzad Baradaran

**Affiliations:** ^1^Department of Biochemistry, Faculty of Medicine, Urmia University of Medical Sciences, Urmia, Iran.; ^2^Cellular and Molecular Research Center, Urmia University of Medical Sciences, Urmia, Iran.; ^3^Immunology Research Center, Tabriz University of Medical Sciences, Tabriz, Iran.; ^4^Department of Immunology, Faculty of Medicine, Tabriz University of Medical Sciences, Tabriz, Iran.; ^5^Pharmaceutical Analysis Research Center, Tabriz University of Medical Sciences, Tabriz, Iran.

**Keywords:** Gastric cancer, siRNA, B7-H7, Docetaxel, Chemo-sensitivity, Combination therapy

## Abstract

**Purpose:**

Despite the high prevalence of gastric cancer (GC), drug resistance is a major problem for effective chemotherapy. B7-H7 is a novel member of the B7 superfamily and is expressed in most common cancers. However, the role of B7-H7 on the aggressiveness of GC and chemosensitivity has remained unknown. Therefore, this study was designed to assess the effect of B7-H7 suppression using small interference RNA (siRNA) in combination with docetaxel on GC cells.

**Methods:**

MTT test was applied to determine the IC50 of docetaxel and the combined effect of B7-H7 siRNA and docetaxel on the viability of the MKN-45 cells. To determine B7-H7, BCL-2, BAX, and caspase-3-8-9 genes expression, qRT-PCR was performed. Furthermore, flow cytometry was applied to evaluate apoptosis and the cell cycle status. Finally, to evaluate the effect of this combination therapy on migratory capacity and colony-forming ability, wound healing assay and colony formation test were employed, respectively.

**Results:**

B7-H7 suppression increased the chemo-sensitivity of MKN-45 cells to docetaxel. The expression of B7-H7 mRNA was reduced after using B7-H7 siRNA and docetaxel in MKN-45 GC cells. Also, B7-H7 suppression alongside docetaxel reduced cell migration and colony formation rate, arrested the cell cycle at the G2-M phase, and induced apoptosis by modulating the expression of apoptotic target genes.

**Conclusion:**

B7-H7 plays a significant role in the chemo-sensitivity and pathogenesis of GC. Therefore, B7-H7 suppression, in combination with docetaxel, may be a promising therapeutic approach in treating GC.

## Introduction

 Gastric cancer (GC) is certainly one of the most common malignant neoplasms encountered in the whole world. Although the prevalence of this cancer has been declining over the past few decades, the number of people who have lost their lives due to GC is high because of patients with this cancer initially do not show any specific symptoms.^1^ Moreover, the average survival rate of GC patients in the advanced stage of the disease is less than 12 months.^2^ Accordingly, identifying particular molecular targets which are directly associated with the metastasis and chemo-sensitivity of GC might be helpful to enhance GC patient outcomes.

 In this regard, targeting the immune checkpoint molecules of the B7 family has emerged as a relatively new strategy against tumors to develop effective and precise therapies.^3,4^ In addition, it is to note that small interfering RNA (siRNA)-based therapy (20-25 base pairs in length) is an efficient technique for cancer treatment that can suppress the expression of the carcinogenic genes.^5^

 B7-H7, also called HHLA2 and B7y, is recognized as a B7 family member of ligands.^6^ According to recent studies, B7-H7 has a dual co-inhibitory and co-stimulatory function in the T cells responses.^7,8^ Therefore, B7-H7 is regarded as an attractive target for immunotherapy of cancers due to the existence of a direct correlation between high levels of B7-H7 expression with the development of tumors and tumorigenesis.^6,9^ Recent data indicated that B7-H7 is overexpressed in tumor cells in different kinds of cancers, such as renal cell carcinoma,^10^ bladder cancer,^11^ colorectal cancer,^12^ lung cancer,^13,14^ osteosarcoma,^15^ breast cancer,^6^ and GC.^16^ Expression of both B7-H7 mRNA and protein is high in the GC tissue. Also, overexpression of B7-H7 has been associated with low overall survival (OS) in GC patients. Therefore, upregulation of B7-H7 expression in GC tissue is regarded as an unfavorable prognostic marker for OS in GC patients.^16^

 As a semi-synthetic analog of taxane, docetaxel is one of the useful chemotherapy drugs for treating patients with GC. Docetaxel suppresses the disassembly of microtubules by inhibiting microtubules’ depolymerization. However, despite its extensive usage, docetaxel resistance hinders efficient treatment for GC.^17^ On the other hand, growing research has presented that combining chemotherapy drugs with siRNA strategy might be a more hopeful modality in cancer therapy which can induce the efficacy of anti-cancer drugs.^18^

 In the current study, to assess whether B7-H7 plays the main role in the development of GC, we used the siRNA strategy as a targeted therapy to suppress the expression of B7-H7 mRNA. Also, combined with docetaxel, we investigated the effects of B7-H7 siRNA-mediated silencing on cell proliferation, apoptosis, cell cycle, colony formation rate, and cell migration in GC cells, which might suggest their combination as a new strategy for the management of GC.

## Materials and Methods

###  Cell culture and cell line selection

 Initially, MKN-45, AGS, and KATO III human GC cell lines were purchased from the Pasteur Institute cell bank (Tehran, Iran). Then, the standard Gibco RPMI-1640 culture media (Gibco, USA, NYC) enriched with 10% fetal bovine serum (FBS; GIBCO, Carlsbad, CA) was applied to maintain the cells. Next, the cultured cells in T25 cell culture flasks were incubated at 37°C in a 95% humidified atmosphere containing 5% CO2. In this study, to choose the proper cell line for the rest of the experiments, the total RNA of three different GC cell lines (MKN-45, AGC, and KATO III) was extracted and the expression of B7-H7 was investigated by qRT-PCR in the aforementioned cell lines that 2^-∆Ct^ method was used to evaluate the expression of targeted gene in GC cell lines.

###  siRNA transfection

 Regarding the higher expression of B7-H7 in MKN-45 cells in comparison with KATO III and AGS cells, MKN-45 cells were selected. Using the Gene Pulser electroporation system (Bio-Rad), the MKN-45 cells were transfected with B7-H7 siRNA (20-80 pmol) (Table 1). After using the electroporation protocol (TC = 12.5 ms and voltage = 130 V), the appropriate amount of cells was seeded in 6, 24, and 96 well plates according to the ongoing test.

**Table 1:** B7-H7 siRNA sequences

**siRNA**	**Sense**	**Antisense**
B7-H7	GCCAAGAAACAGCUUCCCAUAdTdT	UAUGGGAAGCUGUUUCUUGGCdTdT

###  RNA extraction, cDNA synthesis, and qRT -PCR

 To extract total RNA from MKN-45 cells, we applied the Trizol reagent to separate the whole RNA according to the manufacturer’s directions (GeneAll Biotechnology, Korea). Subsequently, the extracted RNA (1 μg) was used to synthesize cDNA using its particular kit (Biofact, Daejeon, South Korea) via BioRad T100 thermal cycler system. Then, to evaluate B7-H7, Caspase3-8-9, BCL-2, and BAX genes expression, StepOne Plus qRT-PCR Device (Applied Biosystems, Foster City, USA) was utilized to carry out real-time PCR. GAPDH was served as an internal control to assess expression of genes. Also, all reactions were triplicated. For qRT-PCR results, 2^-∆∆Ct^ method was utilized to compare the downregulation or upregulation of targeted genes in transfected or treated cells with control cells (control is assumed as 1). Gene-specific primers sets are presented in Table 2.

**Table 2:** Primer sequences

**Target**	**Forward primer**	**Reverse primer**
B7-H7	CTGAAGCAAACATGGACAGG	GTTGACATGAGAGTGAAACG
Caspase-3	CAAACCTCAGGGAAACATTCAG	CACACAAACAAAACTGCTCC
Caspase-8	CGGACTCTCCAAGAGAACAGG	TCAAAGGTCGTGGTCAAAGCC
Caspase-9	CTGTCTACGGCACAGATGGAT	GGGACTCGTCTTCAGGGGAA
BAX	TTTGCTTCAGGGTTTCATCCA	TCTGCAGCTCCATGTTACTGTC
BCl-2	GAGTTCGGTGGGGTCATGTG	CACCTACCCAGCCTCCGTTA
GAPDH	AACATCATCCCTGCCTCTAC	CTGCTTCACCACCTTCTTG

###  MTT cell viability assay

 The MTT assay evaluated the inhibitory concentration causing 50% inhibition (IC50) of docetaxel on MKN-45 cells. For this aim, a total of 1.5 × 10^4^ MKN-45 cells were seeded in 96-well culture plates. Then, the cells were treated with varying amounts of docetaxel and placed in a CO2 incubator overnight. Then, 24 hours after the treatment, MTT (2 mg/mL) was added to each well and cultivated at 37°C for an extra 4 hours. Next, dimethyl sulfoxide (DMSO) (Sigma‐Aldrich) was added to solubilize the products of the insoluble purple formazan. Finally, the optical density of wells was evaluated with an ELISA reader (Sunrise RC, Tecan, Switzerland) at a wavelength of 570 nm and 620 nm as reference.

###  Apoptosis assay

 The effect of B7-H7 suppression in combination with docetaxel on the apoptosis rate of MKN-45 cells was assessed by Annexin-V-FITC and propidium iodide (PI) double staining kit (Exbio, Czech) and flow cytometry instrument. In brief, 3 × 10^5^ MKN-45 cells were seeded into 6-well culture plates following the transfection with B7-H7 siRNA. Then, the cells were grouped by the following order: B7H7 siRNA, docetaxel treated, B7H7 siRNA transfected + docetaxel treated, and control cells and desired groups were treated with docetaxel. Ultimately, flow cytometry (MiltenyBiotec^TM^ FACS Quant 10; MiltenyBiotec, Germany) and FlowJo software (Tree Star, San Carlos, CA) was employed to detect the stained cells and analyze obtained data, respectively.

###  Cell cycle assay

 The flow cytometry was done to study the suppression impact of B7-H7 alongside docetaxel on the cell cycle arrest in MKN-45 cells. First, 3 × 10^5^ MKN-45 cells were seeded into 6-well culture plates following the transfection with B7-H7 siRNA. Then, the cells were grouped by the following order: B7H7 siRNA, docetaxel treated, B7H7 siRNA transfected + docetaxel treated, and control cells. After treatment with docetaxel, the MKN-45 cells were trypsinized and fixed with ethanol (75%) and incubated at -20°C overnight. The next day, PBS was used for washing the cells and they incubated for 30 min at 37 °C after resuspension of cells in PBS comprising 5 mg/mL of RNase. Finally, the cells were stained with DAPI (1 μg/mL) and kept in the darkness for 15-20 min. After this time, the cell cycle phases in each group were determined by flow cytometry instrument (MiltenyBiotecMACS Quant 10), and FlowJo software (Tree Star, San Carlos, CA) was used to analyze the results of this test.

###  Wound-healing assay (Scratch test)

 The wound-healing assay was done to investigate the combined effect of B7-H7 silencing and docetaxel on the migration rate of MKN-45 cells. For this aim, the B7-H7 siRNA transfected MKN-45, and un-transfected cells (5 × 10^5^ cells per well) were seeded in 24-well culture plates. The cells were grouped by the following order: B7-H7 siRNA, docetaxel, combined B7-H7/docetaxel, and control. After 24 hours of transfection, we created a scratch in the center of the wells with the tip of a yellow micropipette to form an open gap. To follow the rate of cell migration in each group into the wound area, we used OPTIKA (Italy) inverted microscope to take different photographs of the wells at the periods of 0, 24, and 48 hours.

###  Colony formation

 The effect of B7-H7 suppression and docetaxel on the colony-forming ability of MKN-45 cells was evaluated by colony formation assay. Hence, B7-H7 siRNA transfected MKN-45, and un-transfected cells were seeded into each well of 6-well plates (the seeding density of 5 × 10^3^ cells per well) and grouped into B7-H7 siRNA transfected group, docetaxel treated group, combination group, and control group. After incubation for two weeks following the colony formation of MKN-45 cells, the staining of colonies in each well was done using 1 ml crystal violet (Sigma Aldrich, USA). The plate remained at room temperature for half an hour. Finally, the photo of the colonies was taken and using ImageJ software (NIH, MD), the number of theirs in each well was counted.

###  Statistical analysis

 The results of the experiments were described as the means ± standard deviation (SD) and the p-value < 0.05 was regarded as statistically significant. The significance of data was assessed using GraphPad Prism v8 (San Diego, California USA, www.graphpad.com) via student t-test and ANOVA one way and two ways.

## Results and Discussion

 Regardless of an overall decreased incidence of GC worldwide, these patients encounter a high mortality rate. In the metastasis phase of GC, applying conventional treatments has no appropriate consequence in managing this malignancy. One of the reasons is the occurrence of cancer cells chemoresistance as an inescapable phenomenon in GC patients.^19^ Hence, there is a requirement to investigate new strategies to improve GC therapy. It is suggested that combined usage of chemotherapy drugs and targeted gene therapy might be an effective strategy to treat cancers.^20,21^ Members of the B7 family immune checkpoint proteins are well-studied as the main regulators involved in the sensitivity of drugs, metastasis, invasion, and tumor growth.^22^ Also, it has been pointed out that the co-inhibitory molecule of the B7 family contributed significantly to inducing the immune-suppressive tumor microenvironment. As the newest B7 family member, B7-H7 was demonstrated to participate in the tumor immune escape and tumorigenesis. Therefore, targeting B7-H7 has been introduced as an exciting strategy for cancer immunotherapy.^23^ However, there has not been any study on the suppression effect of B7-H7 expression in GC until now. Regarding the ability of docetaxel to enhancement of anti-tumor immune response even at the expression level,^24^ in this investigation, we focused for the first time on the combined effect of B7-H7 suppression and docetaxel on cell proliferation, colony formation, apoptosis, cell cycle and cell migration in GC cells.

###  B7-H7 expression was overexpressed in MKN-45 cell line

**Figure 1:**
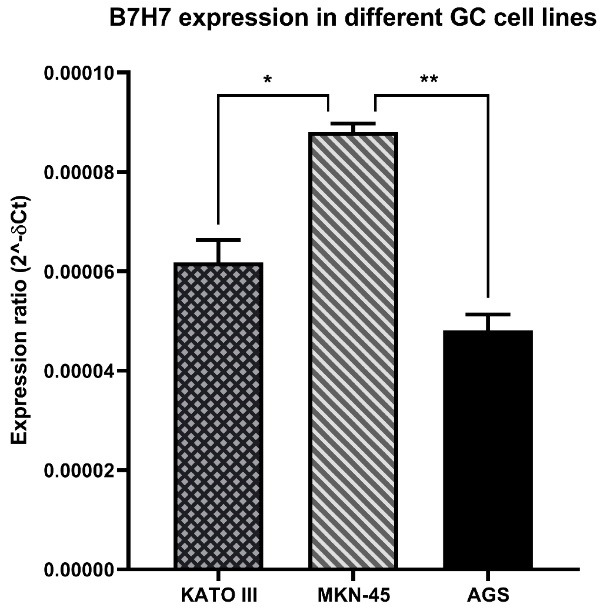
The levels of B7-H7 expression in GC cell lines.The B7-H7 expression was up-regulated in MKN-45 cells compared to KATO III and AGS (*P < 0.05, **P < 0.01)

 Initially, to select the appropriate cell line, the qRT-PCR test was done to assess the expression of B7-H7 in different GC cell lines. According to the obtained results, B7-H7 had high expression levels in the MKN-45 cell line compared to AGS and KATO III cell lines (Figure 1). Consequently, the MKN-45 cell line was chosen for the next upcoming tests in this study.

 In line with our findings, the expression of B7-H7 in GC tissues has been reported previously. A recent study confirmed that B7-H7 is overexpressed in GC tissues in comparison with normal gastric tissue, and B7-H7 expression at high levels indicates a poor OS in these patients.^16^

###  B7-H7 expression was suppressed in MKN-45 cells following specific siRNA transfection

**Figure 2:**
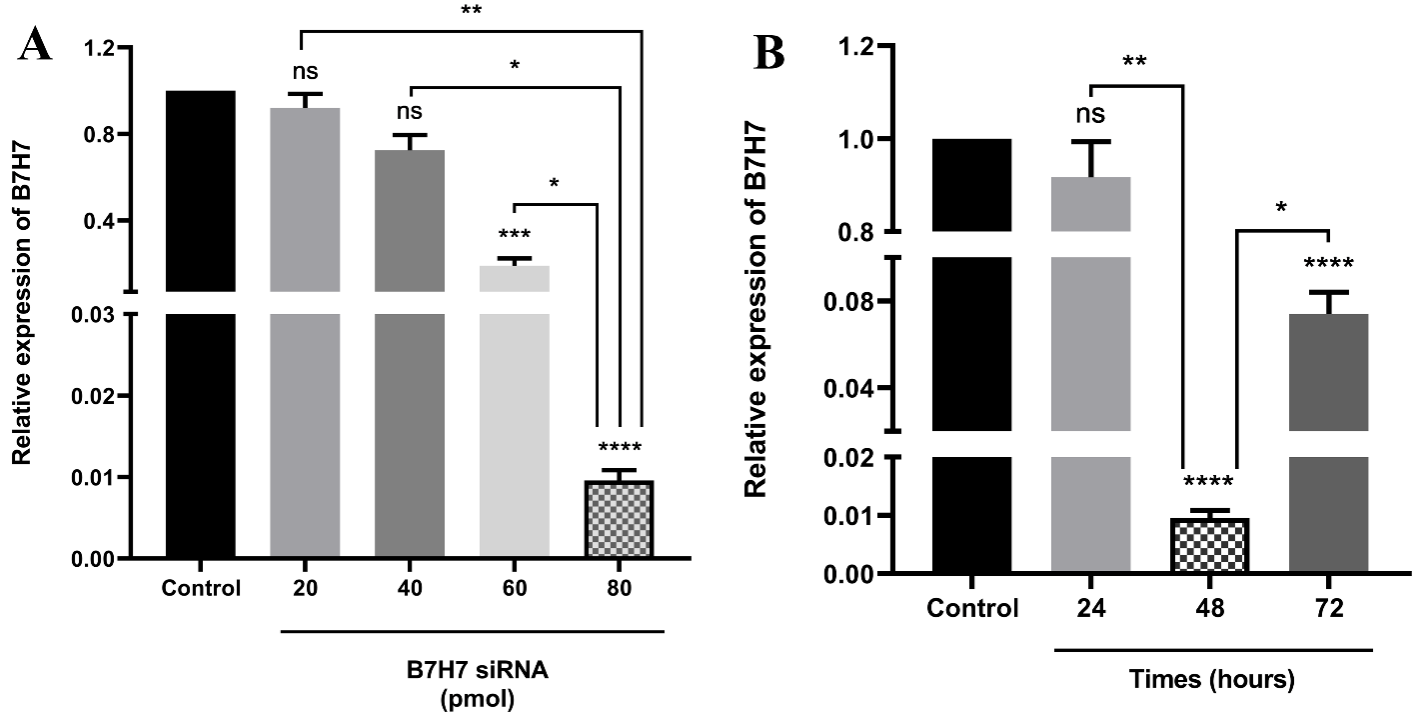
The levels of B7-H7 mRNA expression in MKN-45 cells following transfection of siRNA. **A.** Transfection of B7-H7 siRNA significantly suppressed expression of B7-H7 at the dose of 80 pmol. **B.** B7-H7 siRNA exhibited the highest suppression rate at 48 hours following siRNA transfection compared to 24 and 72 hours (*P <0.05, **P < 0.01, ***P < 0.001, ****P < 0.0001)

 To assess the impact of siRNA, the transfection of MKN-45 cells with various doses of B7-H7 siRNA (20-80 pmol) during different times (24-72 hours) was performed. The analysis of qRT-PCR results revealed that the transfected cells with B7-H7 siRNA had a lower level of B7-H7 expression compared to control cells, and the transfection of MKN-45 cells with 80 pmol dose of B7-H7 siRNA was more efficient in the suppression of B7-H7 expression (Figure 2A). Also, suppression of B7-H7 expression in the steady-state was detected 48 h after the transfection of 80 pmol dose of B7-H7 siRNA (Figure 2B). Consequently, we considered 80 pmol and 48 hours as the optimum dose and the optimum time for the B7-H7 siRNA transfection in MKN-45 cells for the following tests, respectively.

 Chen and colleagues reported that downregulation of B7-H7 expression via RNA interference inhibits the development of ccRCC cells.^25^ Also, Zhang et al showed that silencing of B7H6 as a member of the B7 family by specific siRNA suppresses tumor progression in triple-negative breast cancer.^26^ Moreover, the suppression of B7-H4 by siRNA can considerably suppress the development of HepG2 hepatocellular carcinoma (HCC) cells.^27^ According to the above results, targeting different members of the B7 family via siRNA has shown promising outcomes in the treatment of cancers. However, there has not been any study on the suppression effect of B7-H7 expression in GC until now.

###  B7-H7 siRNA reduced cell viability of MKN-45 GC cells and could increase the sensitivity of MKN-45 cells to docetaxel

**Figure 3:**
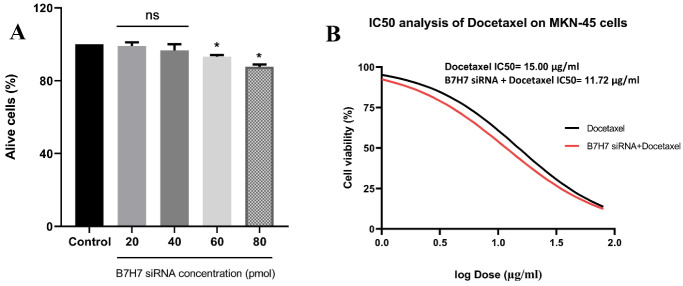
** A.** The effect of B7-H7 siRNA on the viability of MKN-45 cells.Transfection of the individual B7-H7 siRNA resulted in decreased cell viability of MKN-45 cells in a dose-dependent manner (*P < 0.05). **B. **The effect of B7-H7 siRNA alongside Docetaxel on the MKN-45 cells viability.Combined B7-H7 siRNA and Docetaxel usage led to decreased cell viability of MKN-45 cells and IC50 value, indicating that suppression of B7-H7 expression induced chemo-sensitivity of the MKN-45 GC cells to Docetaxel treatment (*P < 0.05)

 MTT test was done to test the effect of various doses of B7-H7 siRNA on the proliferation and viability of MKN-45 cells. Suppression of B7-H7 expression by siRNA decreased the cell viability of MKN-45 cells (Figure 3A). Also, to clarify the impact of B7-H7 siRNA alongside docetaxel treatment on the viability of MKN-45 cells, the MTT test was performed. Transfection of 80 pmol of B7-H7 siRNA alongside different doses of docetaxel caused a significant decrease in MKN-45 cell viability. Besides, suppression of B7-H7 by siRNA decreased the IC50 value of docetaxel. The IC50 value of docetaxel was 15 µg/mL, while the IC50 value of docetaxel in combination with B7-H7 siRNA was 11.72 µg/mL. So, the obtained results revealed that suppression of B7-H7 increases the sensitivity of MKN-45 cells to docetaxel and any change in the viability of MKN-45 cells is mostly related to the docetaxel activity (Figure 3B).

 In the current research, our results demonstrated that the efficient dosage of docetaxel is reduced in combination with B7-H7 siRNA, which means that the chemo-sensitivity of the MKN-45 cells to docetaxel treatment was enhanced by targeting B7-H7. Also, the survival rate of GC cells was low as a consequence of the combined usage of B7-H7 siRNA and docetaxel compared to separate treatment. In accordance with our results, the participation of other members of the B7 family in enhancing the chemo-sensitivity of cancerous cells to chemotherapy agents was illustrated. In this regard, it was indicated that suppression of B7-H3 expression reduces drug resistance of melanoma cells to chemotherapy drugs like Dacarbazine and Cisplatin as well, through inactivation of p38 MAPK and upregulation of DUSP10 expression.^28^ Moreover, a study presented that the downregulation of B7-H6 by shRNA increases the chemo-sensitivity of B-cell lymphoma cells to dexamethasone and vincristine drugs.^29^ Besides, it was revealed that suppression of B7-H3 in breast cancer cells results in improving chemo-sensitivity to paclitaxel through abolishing phosphorylation of Jak2/Stat3.^30^ Consequently, considering our obtaining results, it was hypothesized that B7-H7 might be involved in the chemo-sensitivity of GC cells to docetaxel.

###  B7-H7 suppression combined with docetaxel reduced the expression level of B7-H7 mRNA in MKN-45 cells

**Figure 4:**
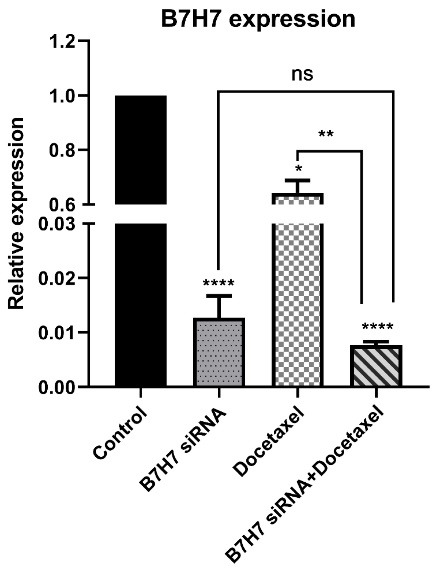
The combination effect of B7-H7 siRNA and Docetaxel on the relative expression of B7-H7 mRNA in MKN-45 cells. As shown in this figure, the change of B7-H7 expression was significant in Docetaxel treated and B7-H7 siRNA transfected groups. However, the combination group suppressed the B7-H7 mRNA expression level more than other groups (*P < 0.05, **P < 0.01, ****P < 0.0001)

 To evaluate the combined effect of B7-H7 suppression by siRNA and docetaxel treatment on the relative expression of B7-H7 in MKN-45 cells, qRT-PCR was performed. The data presented that B7-H7 relative expression was reduced in all experimental groups, while suppression of B7-H7 expression was significant in the B7-H7 siRNA and docetaxel combination groups (Figure 4).

###  Suppression of B7-H7 increased apoptosis rate in MKN-45 GC cells in combination with docetaxel

**Figure 5:**
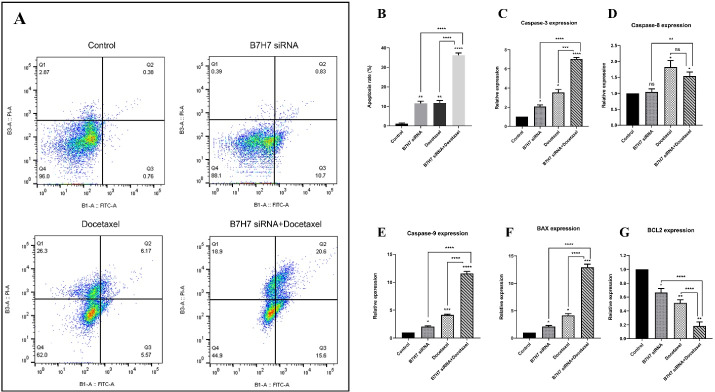
** A, B: **The effect of combination therapy with B7-H7 siRNA and Docetaxel treatment on apoptosis of MKN-45 GC cells. Flow cytometry analysis showed that suppression of B7-H7 in combination with Docetaxel sensitized MKN-45 GC cells to apoptosis. **C, D, E, F, and G. **The combined effect of B7-H7 suppression and Docetaxel treatment on the relative expression of apoptotic target genes. The caspase-3-8-9 and Bax genes expression was increased in the dual usage group compared to the control. Also, suppression of B7-H7 and Docetaxel treatment cooperatively reduced expression of the BCL-2 gene compared to the other groups (*P < 0.05, **P < 0.01, ***P < 0.001, ****P < 0.0001)

 Annexin V/PI staining was utilized to assess the combined effect of B7-H7 suppression and docetaxel treatment on induction of apoptosis in MKN-45 cells. Our findings demonstrated that transfection of cells with B7-H7 siRNA in combination with docetaxel treatment increased apoptosis rate compared to the other groups (control, B7-H7 siRNA, and docetaxel). The apoptosis rate in the combination group was 36.2%, while in the B7-H7 siRNA transfected group and docetaxel treated group were 11.53% and 11.74%, respectively (Figure 5A, B). Furthermore, qRT-PCR was done to confirm the molecular mechanism related to apoptosis induction. For this purpose, we assessed expression levels of BAX and caspase-3-8-9 genes as well as an anti-apoptotic gene like BCL-2 (Figure 5C, D, E, F, G). Our results demonstrated that the combined use of docetaxel and B7-H7 siRNA induced BAX and caspase-3-8-9 expression levels compared to control. Unlike the genes mentioned earlier, the level of Bcl-2 expression as an anti-apoptotic gene was significantly downregulated following combination therapy compared to control and individual treatment.

 The findings of our apoptosis assay indicated that the apoptosis rate was significantly induced in the combination of B7-H7 siRNA and docetaxel. In other words, suppression of B7-H7 using siRNA sensitizes GC cells to the docetaxel through induction of apoptosis. It is noteworthy that caspase-3-8-9, BAX, and BCL-2 genes as key regulators of cell apoptosis were target genes in this investigation. Caspase-3 has a main role in the apoptosis pathway that amplifies the signal together with caspase-8 and caspase-9. Also, The Bax with apoptotic function is the main regulator protein in apoptosis as a vital cellular process. Another target gene was BCL-2 which has an anti-apoptotic function in apoptosis, unlike the BAX gene. Our results demonstrated that suppression of B7-H7 in combination with docetaxel resulted in not only increasing the level of BAX, caspase-3-8-9 genes expression as apoptotic genes but also reducing the level of BCL-2 gene expression as an anti-apoptotic gene. A former study demonstrated that suppression of B7-H6 expression leads to increased expression of BAX and Caspase-3 as well as downregulated BCL-2 expression in triple-negative breast cancer.^26^ Moreover, it was revealed that B7-H3 siRNA in combination with gemcitabine induces apoptosis in pancreatic cancer cells. They indicated that the level of caspase-3-8-9 gene expression is enhanced following B7-H3 silencing combined with gemcitabine treatment, while their expression was not noticeably affected following the suppression of B7-H3 alone in the pancreatic cancer cell line.^31^

###  B7-H7 suppression combined with docetaxel led to MKN-45 cells arrest at the G2-M phase of cell-cycle

**Figure 6:**
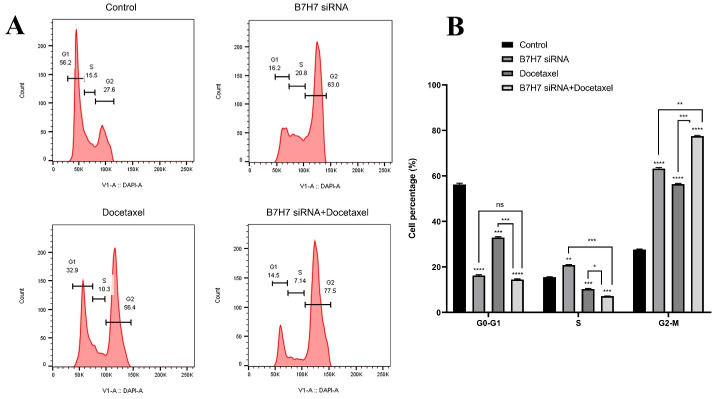
**A, B:** The effect of combination therapy with B7-H7 siRNA and Docetaxel on cell proliferation. B7-H7 siRNA alongside Docetaxel induced G2-M cell cycle arrest in MKN-45 cells (*P < 0.05, **P < 0.01, ***P < 0.001, ****P < 0.0001)

 To examine the effect of individual and combination usage of B7-H7 and docetaxel on the cell cycle progression in MKN-45 cells, the analysis of flow cytometry was done. Compared to the control group, individual usage of B7-H7 suppression and treatment with docetaxel increased the population of G2-M phase cells from 27.6% to 63% and 56.4%, respectively. Interestingly, the highest rate of G2-M arrest has been evaluated in the combined B7-H7 siRNA/docetaxel group (77.5%). Thus, it was demonstrated that dual usage of B7-H7 siRNA and docetaxel could increase the percentage of MKN-45 cells at the G2-M phase and reduce the MKN-45 cell proliferation more than docetaxel and B7-H7 siRNA separately (Figure 6A, B).

 Analysis of our flow cytometry for detection of cell cycle arrest revealed that suppression of B7-H7 expression in combination with docetaxel treatment causes GC cells arrest at the G2-M phase of the cell cycle. Modification of cells could affect any cell cycle checkpoints and inhibit the progression of the cells cycle. One of these cell cycle checkpoints is the Spindle assembly checkpoint located at the end of the mitosis phase. Suppose a modification affects this checkpoint and arrests cell cycle progression. In that case, we can see an increase in the number of cells in the M phase arrested behind the spindle assembly checkpoint, as shown in Figure 6.^32^ Consistent with our findings, to verify the function of B7-H7 on the progression of the cycle in cancerous cells, Chen et al indicated that downregulation of B7-H7 using shRNA in human ccRCC cell lines considerably contributes to inducing the cell cycle arrest at the G1 phase.^25^ Also, it was shown that suppression of B7-H6 could induce apoptosis in human glioma, and the cells within the G1 phase are increased.^33^

###  Suppression of B7-H7 inhibited migration of MKN-45 cells in combination with docetaxel

**Figure 7:**
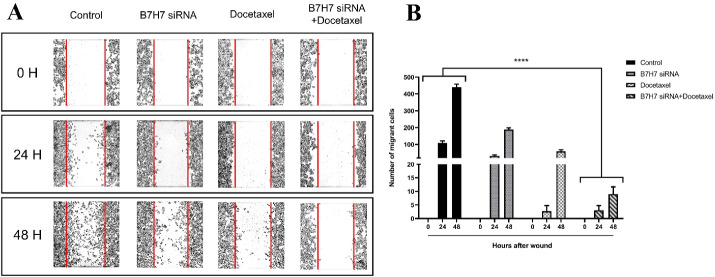
**A, B: **The inhibitory effect of B7-H7 siRNA in combination with Docetaxel on the migration of MKN-45 GC cells. An inverted microscope was used to capture photos of a gap between cells in the control, B7-H7 siRNA, Docetaxel, and a combination of Docetaxel and B7-H7 siRNA groups at the periods of 0, 24, and 48 hours. The number of migrated cells was significantly reduced following combination therapy compared to the control and individual treatments (****P < 0.0001)

 Metastasis and invasion are significant characteristics of tumors that cause the migration of cells. A wound-healing assay was done to determine the migration ability of MKN-45 cells. Our findings demonstrated that the siRNA-mediated knockdown of B7-H7 in combination with docetaxel led to a noticeable anti-migration impact on the migration of the MKN-45 group (Figure 7A, B).

 Our experiment investigated whether the expression of B7-H7 is involved in the migration of GC cells. According to our findings, suppression of B7-H7 or docetaxel treatment alone reduced the migratory property of GC cells; however, the combined usage of B7-H7 siRNA/docetaxel could considerably increase their effects on the inhibition of GC cell migration. Regarding the inhibitory role of B7-H7 suppression on the migration of cancer cells, Chen et al showed that suppression of B7-H7 significantly decreased ccRCC cell migration.^25^ It has been demonstrated that in lymphoma, human glioma tissues, and triple-negative breast cancer, suppression of B7-H6 inhibited cell migration more considerably compared to the control group.^26,29,33^ Besides, the B7-H4 expression was confirmed to be associated with the migration of HCC cells, and its downregulation with siRNA could considerably suppress the progression of HCC cells.^27^

###  B7-H7 suppression and docetaxel cooperatively reduced the clonogenic ability of MKN-45 cells

**Figure 8:**
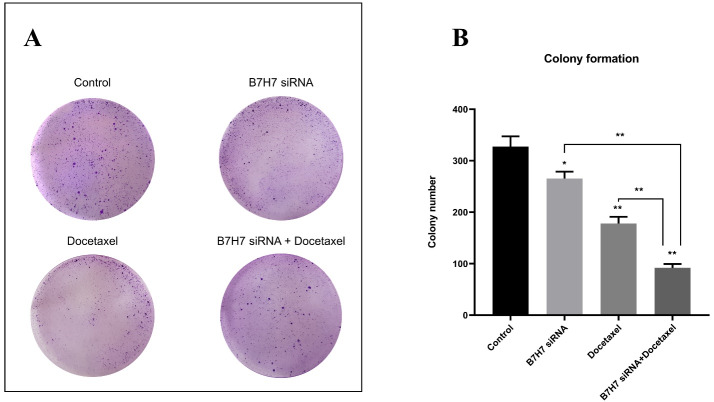
**A, B:** The effect of combination therapy with B7-H7 siRNA and Docetaxel on colony formation of MKN-45 cells. The lowest rate of colony formation was observed in the B7-H7 siRNA/Docetaxel combination group compared to other groups (*P < 0.05, **P < 0.01)

 To perceive the clonogenic ability of MKN-45 cells, a colony formation test was done. According to the number of colonies formed in the culture plate, we realized that the colony numbers were considerably reduced following the usage of B7-H7 siRNA alongside docetaxel compared to the control cells and separate treatments (Figure 8). Consequently, silencing the expression of B7-H7 in combination with docetaxel resulted in the lowest colony formation rate in MKN-45 cells.

 One of the noteworthy findings of our study is that individual usage of B7-H7 siRNA or docetaxel reduced the clonogenic ability of GC cells compared to control. However, the B7-H7 suppression alongside docetaxel could reduce the number and growth of colonies compared to separate treatments, supporting the synergistic effect of B7-H7 siRNA and docetaxel treatment. In accordance with our results, a previous study performed the colony formation assay to assess the suppression effect of B7-H6 expression on the lymphoma cells. Following the knockdown of B7H6 by shRNA, an obvious decrease was seen in the number of lymphoma cell colonies.^29^ Also, it was recently demonstrated that downregulation of B7H3 was able to reduce the colony-forming capacity in melanoma cells in combination with cisplatin and dacarbazine drugs.^28^ Furthermore, Dong and colleagues reported that the reduced clonogenic ability of HepG2 cells is a result of suppression of B7-H4 expression using siRNA in HCC cells.^27^

## Conclusion

 In conclusion, our results showed that B7-H7 represents high expression levels in MKN-45 GC cells. Furthermore, our study indicated that suppression of B7-H7 expression could considerably increase chemo-sensitivity of MKN-45 GC cells to docetaxel treatment, suggesting the importance of B7-H7 in the drug responsiveness of GC cells. Importantly, for the first time, our experimental assays demonstrated that combined usage of B7-H7 siRNA and docetaxel could reduce cell proliferation, colony formation, and migration of GC cells. Also, it was established that combined usage of B7-H7 siRNA and docetaxel was able to arrest the cell cycle and induce apoptosis in GC cells via increasing expression of caspase-3-8-9 and BAX genes and decreasing BCL-2 gene expression. So, the combination of B7-H7 siRNA and docetaxel could be considered an effective therapeutic strategy in emerging novel approaches for the management of GC; demanding more studies will lead to a deeper understanding of this combination therapy for GC.

## Competing Interests

 The authors declare that there is no conflict of interest related to this study.

## Ethical Approval

 The present study was supported by Immunology Research Center,

 Tabriz University of Medical Sciences, Tabriz, Iran and Urmia University of Medical Sciences, Urmia, Iran (IR.UMSU.

 REC.1399.071).

## Funding

 Urmia University of Medical Sciences, Grant/Award Number: IR.UMSU.REC.1399.071.

## References

[1] Machlowska J, Baj J, Sitarz M, Maciejewski R, Sitarz R (2020). Gastric cancer: epidemiology, risk factors, classification, genomic characteristics and treatment strategies. Int J Mol Sci.

[2] Casamayor M, Morlock R, Maeda H, Ajani J (2018). Targeted literature review of the global burden of gastric cancer. Ecancermedicalscience.

[3] Leung J, Suh WK (2014). The CD28-B7 family in anti-tumor immunity: emerging concepts in cancer immunotherapy. Immune Netw.

[4] Ni L, Dong C (2017). New B7 family checkpoints in human cancers. Mol Cancer Ther.

[5] Mansoori B, Sandoghchian Shotorbani S, Baradaran B (2014). RNA interference and its role in cancer therapy. Adv Pharm Bull.

[6] Janakiram M, Chinai JM, Fineberg S, Fiser A, Montagna C, Medavarapu R (2015). Expression, clinical significance, and receptor identification of the newest B7 family member HHLA2 protein. Clin Cancer Res.

[7] Zhao R, Chinai JM, Buhl S, Scandiuzzi L, Ray A, Jeon H (2013). HHLA2 is a member of the B7 family and inhibits human CD4 and CD8 T-cell function. Proc Natl Acad Sci U S A.

[8] Zhu Y, Yao S, Iliopoulou BP, Han X, Augustine MM, Xu H (2013). B7-H5 costimulates human T cells via CD28H. Nat Commun.

[9] Janakiram M, Chinai JM, Zhao A, Sparano JA, Zang X (2015). HHLA2 and TMIGD2: new immunotherapeutic targets of the B7 and CD28 families. Oncoimmunology.

[10] Chen D, Chen W, Xu Y, Zhu M, Xiao Y, Shen Y (2019). Upregulated immune checkpoint HHLA2 in clear cell renal cell carcinoma: a novel prognostic biomarker and potential therapeutic target. J Med Genet.

[11] Lin G, Ye H, Wang J, Chen S, Chen X, Zhang C (2019). Immune checkpoint human endogenous retrovirus-H long terminal repeat-associating protein 2 is upregulated and independently predicts unfavorable prognosis in bladder urothelial carcinoma. Nephron.

[12] Zhu Z, Dong W (2018). Overexpression of HHLA2, a member of the B7 family, is associated with worse survival in human colorectal carcinoma. Onco Targets Ther.

[13] Cheng H, Janakiram M, Borczuk A, Lin J, Qiu W, Liu H (2017). HHLA2, a new immune checkpoint member of the B7 family, is widely expressed in human lung cancer and associated with EGFR mutational status. Clin Cancer Res.

[14] Cheng H, Borczuk A, Janakiram M, Ren X, Lin J, Assal A (2018). Wide expression and significance of alternative immune checkpoint molecules, B7x and HHLA2, in PD-L1-negative human lung cancers. Clin Cancer Res.

[15] Koirala P, Roth ME, Gill J, Chinai JM, Ewart MR, Piperdi S (2016). HHLA2, a member of the B7 family, is expressed in human osteosarcoma and is associated with metastases and worse survival. Sci Rep.

[16] Wei L, Tang L, Chang H, Huo S, Li Y (2020). HHLA2 overexpression is a novel biomarker of malignant status and poor prognosis in gastric cancer. Hum Cell.

[17] Suzuki T, Yoshida K, Wada Y, Hamai Y, Sentani K, Oue N (2007). Melanoma-associated antigen-A1 expression predicts resistance to docetaxel and paclitaxel in advanced and recurrent gastric cancer. Oncol Rep.

[18] Mansoori B, Mohammadi A, Davudian S, Shirjang S, Baradaran B (2017). The different mechanisms of cancer drug resistance: a brief review. Adv Pharm Bull.

[19] Marin JJ, Al-Abdulla R, Lozano E, Briz O, Bujanda L, Banales JM (2016). Mechanisms of resistance to chemotherapy in gastric cancer. Anticancer Agents Med Chem.

[20] Bayat Mokhtari R, Homayouni TS, Baluch N, Morgatskaya E, Kumar S, Das B (2017). Combination therapy in combating cancer. Oncotarget.

[21] Asadzadeh Z, Mansoori B, Mohammadi A, Kazemi T, Mokhtarzadeh A, Shanehbandi D (2021). The combination effect of Prominin1 (CD133) suppression and Oxaliplatin treatment in colorectal cancer therapy. Biomed Pharmacother.

[22] Ye Q, Liu J, Xie K (2019). B7 family proteins in cancer progression: immunological and non-immunological functions. J Cancer Treatment Diagn.

[23] Zou W, Chen L (2008). Inhibitory B7-family molecules in the tumour microenvironment. Nat Rev Immunol.

[24] Millrud CR, Mehmeti M, Leandersson K (2018). Docetaxel promotes the generation of anti-tumorigenic human macrophages. Exp Cell Res.

[25] Chen L, Zhu D, Feng J, Zhou Y, Wang Q, Feng H (2019). Overexpression of HHLA2 in human clear cell renal cell carcinoma is significantly associated with poor survival of the patients. Cancer Cell Int.

[26] Zhang B, Sun J, Yao X, Li J, Tu Y, Yao F (2018). Knockdown of B7H6 inhibits tumor progression in triple-negative breast cancer. Oncol Lett.

[27] Dong L, Xie L, Li M, Dai H, Wang X, Wang P (2019). Downregulation of B7-H4 suppresses tumor progression of hepatocellular carcinoma. Sci Rep.

[28] Flem-Karlsen K, Tekle C, Øyjord T, Flørenes VA, Mælandsmo GM, Fodstad Ø (2019). p38 MAPK activation through B7-H3-mediated DUSP10 repression promotes chemoresistance. Sci Rep.

[29] Wu F, Wang J, Ke X (2016). Knockdown of B7-H6 inhibits tumor progression and enhances chemosensitivity in B-cell non-Hodgkin lymphoma. Int J Oncol.

[30] Liu H, Tekle C, Chen YW, Kristian A, Zhao Y, Zhou M (2011). B7-H3 silencing increases paclitaxel sensitivity by abrogating Jak2/Stat3 phosphorylation. Mol Cancer Ther.

[31] Wang C, Liu Y, Zhuang X, Xu C (2016). Knock-down of B7-H3 by small interfering RNA promotes gemcitabine-induced apoptosis in pancreatic cell line Patu8988t. Int J Clin Exp Med.

[32] Musacchio A, Salmon ED (2007). The spindle-assembly checkpoint in space and time. Nat Rev Mol Cell Biol.

[33] Jiang T, Wu W, Zhang H, Zhang X, Zhang D, Wang Q (2017). High expression of B7-H6 in human glioma tissues promotes tumor progression. Oncotarget.

